# Case report: Steroid-responsive acute chorea as first presentation of the coexistence of Moyamoya and Graves' disease

**DOI:** 10.3389/fneur.2023.1170837

**Published:** 2023-06-28

**Authors:** Wei-Sheng Wang, Shey-Lin Wu, Wei-Chieh Chan, Yen-Chung Chen

**Affiliations:** ^1^Department of Neurology, Changhua Christian Hospital, Changhua, Taiwan; ^2^Department of Electrical Engineering, National Changhua University of Education, Changhua, Taiwan; ^3^Department of Public Health, Chung Shan Medical University, Taichung, Taiwan

**Keywords:** chorea, hyperthyroidism, Graves' disease, Moyamoya disease, cerebral perfusion scan

## Abstract

**Background:**

Chorea is a movement disorder characterized by abrupt, rapid, and uncontrollable, random movements from one part of the body to another with motor impersistence. Sporadic chorea is rarely caused by either thyrotoxicosis or Moyamoya disease (MMD).

**Methods and results:**

In this case report, we describe a female patient with chorea with the rare coexistence of Graves' disease and Moyamoya disease. Tc-99m ethyl cysteinate dimer (ECD) brain perfusion single-photon emission computed tomography (SPECT) showed mild to moderate hypoperfusion in bilateral frontal and left temporal regions. After administering dexamethasone 20 mg for 5 days, her choreic movement symptoms recovered rapidly.

**Conclusion:**

Although uncommon, thyrotoxicosis and Moyamoya disease can co-occur, especially in Asian female adults. Excessive thyroid hormones contribute to the dysregulation of neurotransmitters in basal ganglia-thalamocortical circuits. Moyamoya disease is responsible for ischemic changes affecting the excitatory–inhibitory circuits between the basal ganglia and the neocortex. Under a state of coexistence, thyrotoxicosis exaggerates cerebral metabolism, aggravating the impaired cerebral perfusion induced by Moyamoya disease. Moreover, inflammatory reactions caused by thyroid autoantibodies may also promote the progression of Moyamoya disease. In our experience, treatment with steroids may not only synergize the anti-thyroid effect but may also be a way to modulate the neurotransmitters within the basal ganglia or restore cerebral perfusion. We suggest that evaluation of the thyroid function status in Moyamoya disease is essential.

## Introduction

Thyrotoxicosis and Moyamoya disease (MMD) are both rare causes of sporadic chorea, accounting for 2% and 3–6% of cases, respectively (1–3). Although uncommon, thyrotoxicosis and Moyamoya disease sometimes appear to coexist, especially in Asian female adults (4). Excessive thyroid hormones contribute to the dysregulation of neurotransmitters in basal ganglia-thalamocortical circuits (5). Moyamoya disease is responsible for ischemic changes affecting the excitatory–inhibitory circuits between the basal ganglia and the neocortex (6). In management, despite medical correction of the state of thyrotoxicosis, a timely combination of steroids may not only synergize the anti-thyroid effect but also may be a possible curative treatment for involuntary movement disorders (7–10). In this study, we present an adult case of progressive chorea with the coexistence of Graves' disease and Moyamoya disease and demonstrate the therapeutic effect of short-term steroid use.

## Case presentation

A 35-year-old woman presented to the clinic with progressive, left-sided dominant, fluid, unpredictable but sometimes suppressible, dance-like movements. She stated that the symptoms, which had been present for 1 year, originated in her left limbs, resulting in mild and occasional difficulty with gait, swallowing, and speech, which impeded her daily life. However, over the preceding 2 weeks, the symptoms had spread to her right limbs, trunk, and cervical area. Due to her persistent choreoathetosis, she was unable to adequately perform her job as a construction worker.

The patient had a past surgical history of rhinoplasty after a traffic accident and cholecystectomy due to cholecystitis 2 years prior. She had never taken neuroleptic medications, had no history of diabetes mellitus, hypertension, or hypoxic injury, and had no family history of psychiatric illness or movement disorders. She denied having a fever, altered mental status, headache, dizziness, double vision, limb weakness, muscle cramping, weight loss, medication use, or any skin/joint lesions during the whole course of treatment.

A neurological examination revealed that she was lucid and alert. Bilateral muscle powers were full with no sensory deficiency. There were no pathological reflexes or focal signs in the cranial nerves. In addition to paroxysmal choreoathetosis of the limbs, trunk, and cervical spine, motor impersistence was also observed in the left limbs. In coordination, dysdiadochokinesia and mild dysmetria were noted, especially over the left limbs; the patient presented with a dystonic gait. There was no rigidity or spasticity, but the patient tended to display antecollis (Supplementary Video 1, before).

After admission, blood and urine specimens were obtained for routine laboratory analysis, all of which were within normal limits. Autoimmune titers and serologic screening for human immunodeficiency virus and syphilis were also performed. An elevated free T4 level with undetectable TSH was noted. Furthermore, anti-TSH receptor antibodies and anti-thyroid peroxidase antibodies were both positive (Table 1). Ultrasonography of the thyroid revealed bilateral diffuse goiters, which was compatible with Graves' disease.

**Table 1:** Thyroid function status and autoantibodies.

TSH	< 0.005 (L)	uIU/ml
Free T4	5.35 (H)	ng/dl
T3	5.47 (H)	ng/ml
Intact PTH	41.8	pg/ml
Anti-TSH receptor antibody	13.99 (H)	IU/l
Anti-TPO	277.5 (H)	IU/ml
Thyroglobulin antibody	7.9 (H)	IU/ml

**Figure 1:**
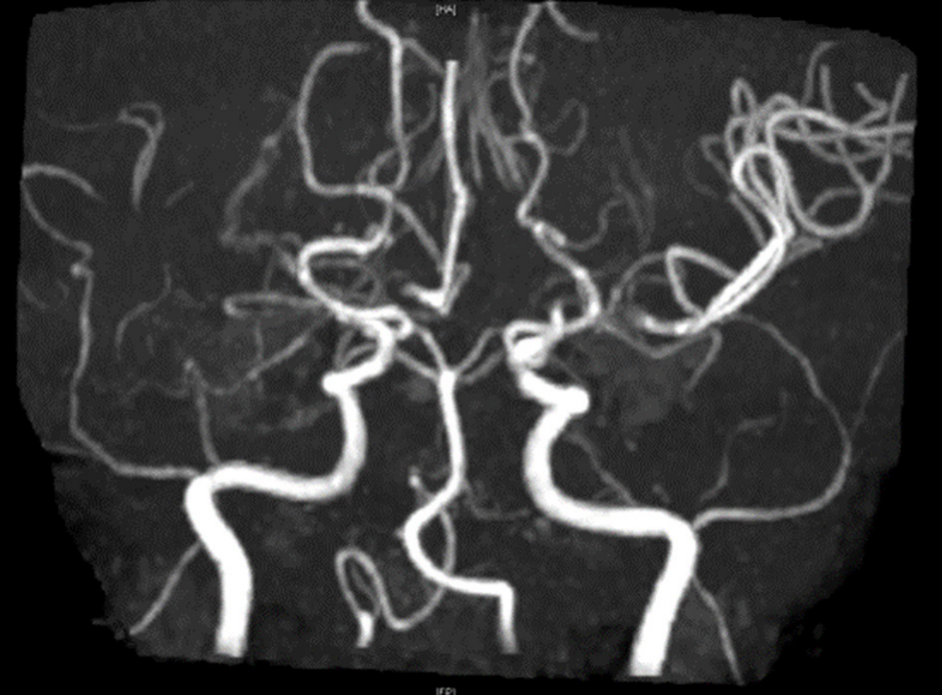
MR angiography shows multiple levels of abnormal progressive vessel luminal stenoses involving bilateral ICA supraclinoid segments, bilateral ACA A1 segments, and bilateral MCA M1 segments, which implied Moyamoya disease.

**Figure 2:**
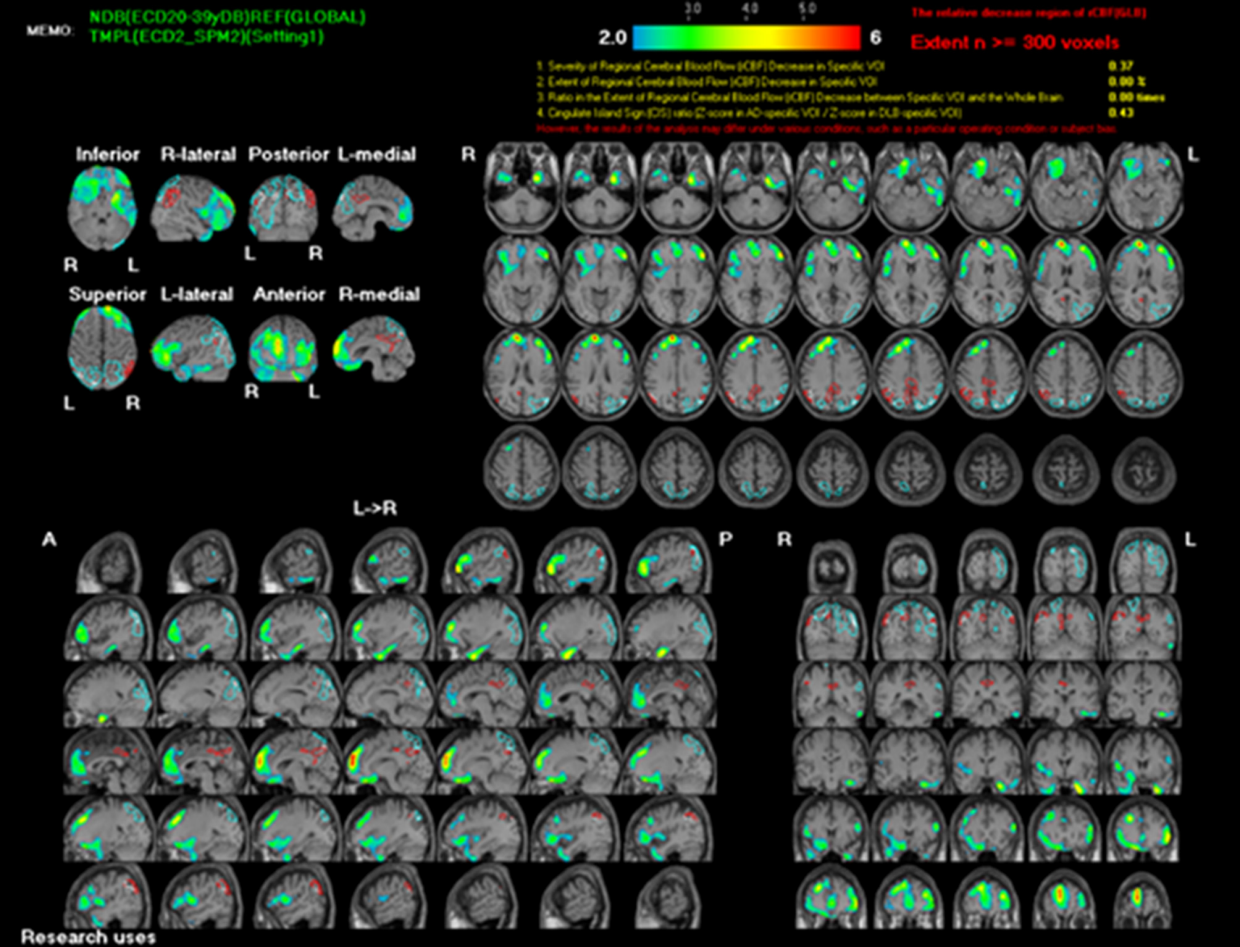
Tc-99m ECD brain SPECT scan shows mild to moderate hypoperfusion in bilateral frontal and left temporal regions.

To rule out structural lesions, a brain MRI with contrast was also performed. The MRI showed multiple levels of abnormal progressive luminal vessel stenosis involving bilateral ICA supraclinoid segments, bilateral ACA A1 segments, and bilateral MCA M1 segments (Figure 1). The Moyamoya disease was impressed. Tc-99m ECD brain perfusion SPECT was also carried out to evaluate cerebral circulation, which showed mild to moderate hypoperfusion in bilateral frontal and left temporal regions (Figure 2). CSF analysis was within normal limits. After administering 20 mg dexamethasone for 5 days, the patient's symptoms of paroxysmal choreoathetosis and dystonia recovered rapidly (Supplementary Video 1, after). The long-term use of methimazole was recommended, and the patient was then referred to a neurosurgeon for further surgical intervention of revascularization.

## Discussion

Thyrotoxicosis accounts for < 2% of cases of chorea (1). Although the mechanism is still not well understood, possible hypotheses include striatal dysfunction, hyperactivity of dopamine receptors, and hyperadrenergic state with dysregulation of the sympathetic nervous system (11). The level of homovanillic acid (HVA), a metabolite of dopamine in the CSF, is reduced in patients with thyrotoxicosis victims, suggesting hyperactivity of dopamine receptors resulting in a decrease in dopamine turnover (12). Experimental guinea pigs with induced thyrotoxicosis have been observed to have increased dopaminergic receptor sensitivity and decreased catecholamine turnover: consequently, it has also been proposed that thyrotoxicosis-induced chorea may be blamed for increased sensitivity of striatal catecholamine receptors (5). Cerebral perfusion abnormalities in chorea have also been recorded, and while some reported hypoperfusion as in our case, others reported hyperperfusion. The affected cerebral regions varied as well. Further studies, especially with longitudinal follow-up, are needed to clarify the relationship (13, 14).

On the other hand, the cerebral angiopathy - Moyamoya disease (MMD), characterized by progressive narrowing of the intracranial carotid artery, then developing collateral vessels by compensation with the aspect of a “puff of smoke”, may indeed be the occurrence of chorea properly (15). Clinically, however, MMD is more associated with ischemic events, particularly in pediatrics; presentation with movement disorders is rare, with an estimated frequency of 3–6% (2, 3). Its pathophysiological mechanism is still poorly understood; some have suggested that the choreoathetosis in MMD is a result of active ischemic changes affecting the excitatory–inhibitory circuits between the basal ganglia and the neocortex (6). A small literature review published in Frontiers in Neurology (16) revealed that hypermetabolism noticed in the pathway of basal ganglia-thalamocortical circuits using an 18F-FDG PET may explain the MMD-induced chorea, rather than the striatal hypoperfusion previously detected by SPECT (16).

In addition, thyrotoxicosis and MMD can be concurrent, especially in Asian adult females. Under the state of thyrotoxicosis, excessive thyroid hormones have been shown to stimulate cerebral metabolism and oxygen consumption, aggravating the impaired cerebral perfusion induced by MMD (4). Furthermore, thyroid autoantibodies have been shown to be independently associated with the risk and prognosis of MMD; another hypothesis is that the immune and inflammatory reaction may also contribute to the simultaneous presentation of thyrotoxicosis and MMD (17, 18).

Because thyrotoxicosis may promote the progression of vasculopathy, medical correction of thyrotoxicosis should be considered first-line treatment. Non-compliance should be avoided because thyroid function may be impaired, possibly resulting in a relapse of associated neurological symptoms and triggering more ischemic events (19). A timely combination of steroids may not only dampen thyrotoxicosis but also represent a possible curative treatment for involuntary movement disorders by modulating neurotransmitters within the basal ganglia or restoring cerebral perfusion (7–10). The role of surgical intervention with revascularization is poorly understood; some clinicians reserve surgery for patients suffering from persistent or worsening neurological symptoms even with prompt medical therapy. Even with surgery, maintaining normal thyroid function is critical to avoid the recurrence of both symptoms and vascular abnormalities (20).

## Conclusion

To the best of our knowledge, this is a rare report of a patient with chorea and co-occurrence of Graves' disease and MMD. Thyrotoxicosis and chorea were successfully treated with short-term dexamethasone (20 mg for 5 days). Steroid treatment could represent a bridge therapy before surgical revascularization. In similar scenarios, particularly in Asian women, resolution of thyrotoxicosis in MMD is essential for timely relief of involuntary movements, better management of cerebral hemodynamic abnormalities, and even prevention of further devastating ischemic events.

## Data availability statement

The original contributions presented in the study are included in the article/Supplementary material, further inquiries can be directed to the corresponding author.

## Ethics statement

Ethical review and approval was not required for the study on human participants in accordance with the local legislation and institutional requirements. Written informed consent from the patients/participants or patients/participants' legal guardian/next of kin was not required to participate in this study in accordance with the national legislation and the institutional requirements. Written informed consent was obtained from the individual(s) for the publication of any potentially identifiable images or data included in this article.

## Author contributions

W-SW collected the case history, figures, and then composed the manuscript with literature review. W-CC provided in-hospital care and conducted written informed consent. S-LW critically reviewed and edited the manuscript. Y-CC conceptualized the manuscript and is the main guarantor of this work. All authors contributed to the article and approved the final version to be published.
